# High-Strength Steel in Civil Engineering Structures: A Review of Material Behaviour, Durability, Fatigue and Component Performance

**DOI:** 10.3390/ma19163509

**Published:** 2026-08-19

**Authors:** Ziheng Ding, Xuanyi Xue, Fei Wang, Neng Wang, Shuai Li, Jianmin Hua

**Affiliations:** 1 School of Civil Engineering, Chongqing University, Chongqing 400045, China27130461@alu.cqu.edu.cn (F.W.);; 2 State Key Laboratory of Safety and Resilience of Civil Engineering in Mountain Area, Chongqing 400045, China; 3 School of Management Science and Real Estate, Chongqing University, Chongqing 400045, China; 4 Department of Civil Engineering, The University of Hong Kong, Pokfulam Road, Hong Kong, China

**Keywords:** high-strength steel, civil engineering, mechanical behaviour, durability, component performance

## Abstract

High-strength steel has attracted increasing attention in civil engineering because of its high strength-to-weight ratio and potential for material-efficient design. This narrative review, supported by a structured literature search, summarizes recent advances in the material behaviour, durability and structural performance of high-strength steel. The discussion covers constitutive behaviour, fatigue and fracture, corrosion degradation, high-temperature and post-fire properties, residual stresses, structural members and connections. Existing studies show that increasing steel strength is commonly accompanied by reduced ductility and strain-hardening capacity, while local buckling, residual stress, welding-induced heterogeneity, fatigue damage, corrosion and thermal degradation remain important design concerns. The accuracy of current design provisions varies with steel grade, product form, section geometry, failure mode and exposure condition, and direct extension from conventional steels is not always appropriate. Future research should emphasize coupled degradation mechanisms, consistent material characterization, broader experimental validation and design models with clearly defined applicability limits.

## 1. Introduction

Steel structures are widely used in buildings, bridges, offshore platforms and industrial facilities. Their advantages include high load-bearing capacity, reliable prefabrication and rapid construction. However, conventional structural steels are increasingly challenged by the demand for longer spans, taller buildings, lighter members and lower carbon emissions. These requirements have promoted the use of high-strength steel (HSS) in civil engineering structures.

HSS is commonly defined as structural steel with a nominal yield strength not lower than 460 MPa [1,2,3]. Compared with conventional structural steel, HSS can provide higher load resistance with smaller member sizes. This can reduce material consumption, welding volume, transportation demand and foundation load. It is therefore attractive for high-rise buildings, long-span structures, bridges and weight-sensitive structural systems [1,2,4]. Recent studies also indicate that the increase in carbon footprint associated with higher steel grades can be limited for some product categories. Thus, the material saving achieved by HSS may contribute directly to lower embodied carbon in suitable applications [1]. The application of HSS is also supported by advances in steelmaking and forming technologies. Quenching and tempering, thermo-mechanical controlled processing, cold forming and welding techniques have enabled the production of structural steels with improved strength, toughness and weldability [2,4]. HSS plates, welded sections, hot-finished tubes and cold-formed hollow sections have been adopted in a wide range of structural applications. These developments have created new opportunities for efficient structural design. However, the benefits of HSS are not universal. They depend on member slenderness, section type, fabrication route, connection detail and loading condition [1,4].

The mechanical behaviour of HSS differs from that of conventional structural steel. With increasing steel grade, the yield-to-tensile strength ratio usually increases, while elongation and strain-hardening capacity tend to decrease [2,4]. A distinct yield plateau is often absent in many HSS products. The 0.2% proof stress is therefore commonly used in design. These features affect plastic redistribution, ductility demand, fracture resistance and seismic energy dissipation. They also limit the direct extension of material models and design assumptions developed for mild steel [2,4]. Durability and service-induced degradation introduce further uncertainty. HSS structures may be exposed to corrosion, cyclic loading, impact, fire and low-temperature environments during service. Corrosion pits can cause local stress concentration and accelerate crack initiation. Fatigue and pre-fatigue damage can reduce residual strength, ductility and energy dissipation capacity. Fire exposure and post-fire cooling can alter strength, ductility and fracture behaviour. These degradation mechanisms are often coupled. They are also sensitive to steel grade, microstructure, welding residual stress, surface condition and environmental history [5,6,7,8].

At the structural level, many studies have examined HSS columns, beams, hollow sections, plate elements and connections. The results generally demonstrate the potential of HSS for achieving high structural efficiency. However, local buckling, global instability, residual stresses, heat-affected-zone softening and limited deformation capacity remain important concerns [1,2,4]. Current design standards have gradually expanded their applicable strength ranges. Nevertheless, many provisions are still based on ordinary-strength steel data. Their accuracy may be insufficient for ultra-high-strength steel (UHSS), cold-formed products, tubular joints, complex loading paths and coupled environmental actions [2,3,4]. Recent reviews have addressed steel-grade selection, hollow sections, codification, product standards, corrosion and fatigue [1,2,3,4,5,6,7,8]. However, most focus on individual topics, and the links among material behaviour, environmental degradation, member response and connection design remain only partly synthesized. The present review therefore provides a narrative overview across these areas, with attention to the reported applicability limits of existing models and design provisions.

This article adopts a narrative review approach supported by a structured literature search. Searches were conducted in the Web of Science, Scopus, and ScienceDirect and were last updated in Jun 2026. The searches were limited to English-language journal articles and combined “high-strength steel” or “ultra-high-strength steel” with terms including “stress–strain”, “constitutive model”, “cyclic behaviour”, “fatigue”, “fracture”, “corrosion”, “fire”, “post-fire”, “residual stress”, “column”, “beam”, “beam-column”, “plate girder”, “welded connection”, “bolted connection”, and “tubular joint”. Studies were prioritized when the steel grade, product form, loading or exposure condition, and principal material or structural response could be identified. Research directly related to civil structural behaviour formed the core evidence base. Selected studies from adjacent fields were also retained as supporting references when they provided relevant information on manufacturing processes, hot-deformation behaviour, constitutive modelling, microstructural evolution, or interface-related mechanisms. 

This review examines the behaviour of structural HSS from the material to the component level. For consistency in this article, HSS denotes structural steel with nominal yield strength not below 460 MPa, while UHSS denotes grades with nominal yield strength of 690 MPa or higher. Table 1 summarizes the representative grades and specifications of HSS. The thematic blocks were selected to follow the progression from constitutive and degradation mechanisms to residual stresses, member behaviour and connection performance, as these aspects directly affect structural resistance and the applicability of current design provisions. The main focus is on structural steel materials and isolated steel components. Full structural systems, steel–concrete composite systems, construction methods, and life-cycle or economic assessment are not considered as separate themes.

## 2. Material Behaviour of High-Strength Steels

### 2.1. Stress–Strain Behaviour and Constitutive Modelling

**Figure 1:**
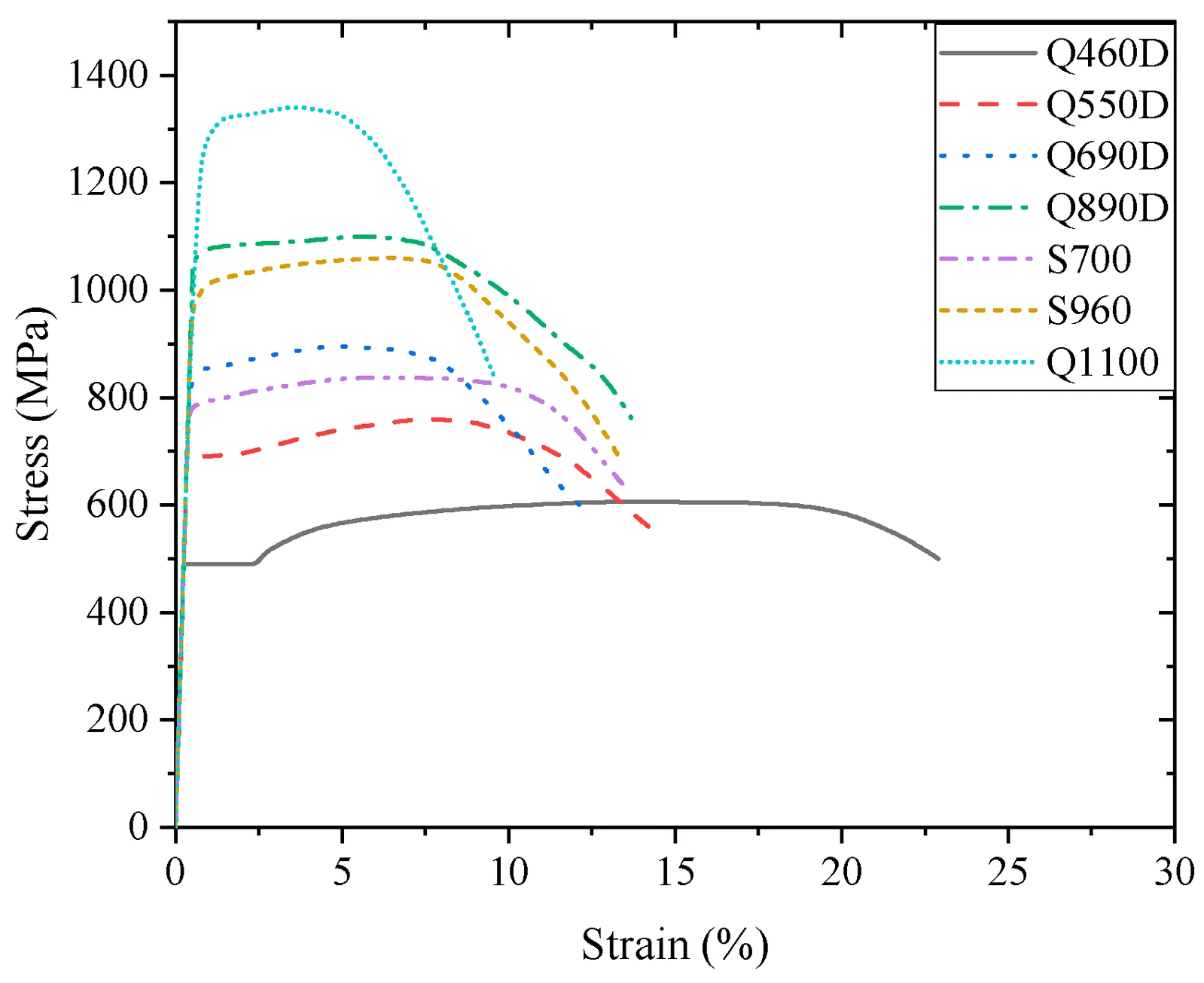
Representative engineering stress–strain curves of HSS with different nominal grades. Adapted from Refs. [10,15,21].

High-strength structural steels generally exhibit higher yield-to-tensile strength ratios and lower elongation than conventional structural steels, although adequate toughness can still be achieved. This feature limits the direct extension of design assumptions developed for mild steels, especially when plastic redistribution, ductility demand, and seismic energy dissipation are involved [9,10]. These differences are reflected in the representative stress–strain curves in Figure 1. Their elastic moduli were close to those of conventional steels, but the yield plateau became shorter and the strain-hardening reserve decreased with increasing steel grade [10]. Existing models based mainly on conventional steels cannot fully capture the yield plateau, strength softening, and loading-history dependence of HSS. An HSS model was therefore proposed by introducing a yield plateau and three-stage strength softening into a combined isotropic–kinematic hardening framework [11]. The limitations of a conventional von Mises description are evident under multiaxial loading. Tests on Q550, Q690 and Q890 steels covered stress triaxialities from approximately −1.314 to 1.026 and Lode angles from 0 to π/3 [12]. The von Mises model overpredicted the pure-shear response by approximately 14%, 12% and 13% for Q550, Q690 and Q890, respectively. The corresponding compression errors were approximately 3%, 7% and 5%. Constitutive modelling has further progressed from uniaxial calibration to stress-state-dependent degradation. The three-dimensional HSS constitutive model incorporated Lode-angle-dependent yielding, combined hardening, three-stage strength softening, and damage accumulation [13]. Beyond ambient-temperature behaviour, the strain rate and service environment can significantly affect the stress–strain response, indicating the need for environment-dependent constitutive models in fire, impact, and polar applications [14,15]. In general, constitutive models calibrated from uniaxial monotonic or cyclic data are efficient for routine structural analysis, whereas stress-state-dependent formulations are more suitable when multiaxial yielding, damage or fracture is important [11,13]. Their applicability remains dependent on the loading path, strain rate, temperature and calibration range of the underlying tests [13,14,15].

Manufacturing route is an additional source of variability in material response and constitutive modelling. Quenching and tempering (QT), thermomechanically controlled processing (TMCP), and direct quenching (DQ) can lead to different sensitivities to welding thermal cycles. Physical thermal simulations of Q690 and Q960 steels showed that the mechanical properties varied across heat-affected-zone (HAZ) subregions [16]. For QT products, low heat input can limit HAZ softening, whereas more pronounced strength reductions have been reported for some TMCP and DQ grades [17]. Cold forming can increase corner strength but may also introduce residual stresses and reduce local ductility [4,18]. Cross-disciplinary hot-deformation studies on X153CrMoV12 tool steel and a lean medium-manganese steel used Arrhenius-type or Zener–Hollomon formulations to describe temperature- and strain-rate-dependent flow behaviour and microstructural evolution [19,20]. These approaches may also provide methodological references for process-sensitive modelling of structural HSS, provided that their applicability is evaluated for the relevant steel grade and manufacturing route.

### 2.2. Cyclic and Fatigue Behaviour

The cyclic response of HSS cannot be inferred directly from its monotonic stress–strain behaviour. Under repeated plastic loading, HSS commonly exhibits the Bauschinger effect, loading-history dependence, and strength degradation. This tendency becomes more pronounced as the strength grade increases. Studies on Q460C mainly supported simplified kinematic hardening descriptions, whereas studies on Q690D, grade 800/1200 steels and Q1100 UHSS showed that cyclic softening should be explicitly considered in constitutive modelling [22,23,24,25]. This suggests that monotonic material models are insufficient for seismic or repeated-loading analysis of HSS structures. Combined isotropic–kinematic hardening models are therefore more appropriate when cyclic degradation and energy dissipation are important.

A further limitation of conventional cyclic models is that many of them are calibrated from uniaxial coupons. Recent studies indicate that cyclic plasticity and fracture of HSS are also stress-state dependent. For Q690 HSS, the conventional definitions of stress triaxiality and Lode angle parameter were found to be inadequate under cyclic loading because backstress modifies the effective stress state [26]. Ductile fracture studies on Q550, Q690, Q890 and Q960 steels further confirmed that fracture prediction should consider both stress triaxiality and Lode angle effects, rather than relying only on uniaxial elongation or triaxiality-based criteria [27,28]. These findings indicate a clear shift from simple cyclic curve fitting toward stress-state constitutive and fracture frameworks.

Fatigue damage provides another link between material degradation and structural performance. Low-cycle pre-fatigue tests on Q690 steel showed that accumulated cyclic damage reduces residual strength and ductility, and that residual stress–strain curves should be described using damage-dependent constitutive models [29]. For high-cycle fatigue, deterministic stress-life curves remain useful, but they cannot fully describe progressive damage evolution. Recent work therefore combined ultrasonic damage indices with inverse Gaussian degradation processes to predict fatigue life and residual life of Q690-series base metal and welded specimens [30]. This trend shows that fatigue assessment is moving from life fitting toward damage process modelling.

Surface damage further complicates fatigue behaviour. Corrosion-fatigue tests on Q690D steel were conducted under constant-amplitude sinusoidal loading at a stress ratio of *R* = 0.1 and a frequency of 30 Hz [31]. Based on the 95% confidence S–N curves and a run-out criterion of 2 × 10^6^ cycles, the fatigue limit decreased from 233.28 MPa for uncorroded specimens to 142.54 MPa after 100 days of simulated marine splash-zone exposure, corresponding to a reduction of 38.89%. The 100-day exposure produced a mass-loss ratio of 7.21% and a maximum pit depth of 510 μm. Corrosion pits and surface roughness act as local stress concentrators, reducing low-cycle fatigue life, accelerating crack initiation and increasing fatigue crack growth rate [32,33]. In this context, average mass loss is insufficient to evaluate fatigue deterioration. Local descriptors such as pit depth, surface roughness and crack-growth threshold are more directly related to fatigue damage.

For offshore and marine applications, multiaxial fatigue should also be considered. Axial–torsional tests on EQ56 marine HSS showed that strain ratio affects cyclic hardening/softening, fatigue life and fracture mode, and that modified critical-plane models can improve life prediction when multiaxial strain-ratio effects are included [34]. Overall, the available literature suggests that reliable cyclic and fatigue modelling of HSS should integrate cyclic softening, stress-state effects, local surface damage and probabilistic degradation processes. Representative studies on cyclic behaviour, fatigue and fracture are summarized in Table 2.

### 2.3. Corrosion and Environmental Degradation

**Figure 2:**
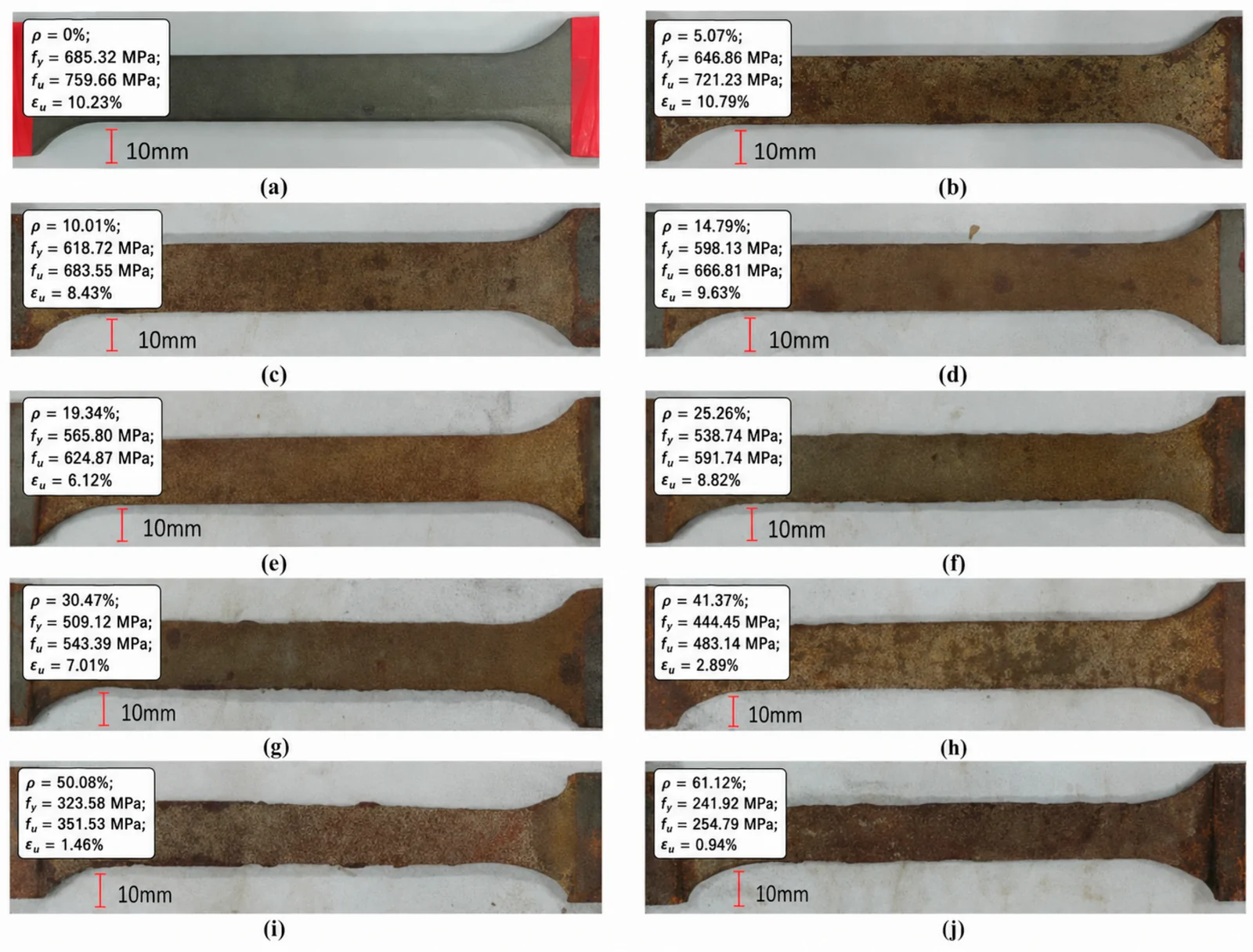
Evolution of corrosion morphology of Q620 HSS specimens with increasing mass loss ratio: (**a**) uncorroded; (**b**) 5.07%; (**c**) 10.01%; (**d**) 14.79%; (**e**) 19.34%; (**f**) 25.26%; (**g**) 30.47%; (**h**) 41.37%; (**i**) 50.08%; (**j**) 61.12%. Adapted from Xue et al. [36].

Related studies on corrosion degradation of HSS are summarized in Table 2. Across electrochemical corrosion, salt-spray exposure, periodic corrosion exposure and flowing seawater tests, the main degradation feature was the gradual transition from relatively uniform surface attack to localized pitting, surface roughening and corrosion layer detachment [35,37,38,39]. This point is particularly important for HSS. Their higher strength is often achieved with lower ductility and thinner plates. Local corrosion defects can therefore reduce deformation capacity and fracture resistance even when the average mass loss is still moderate [36,37]. Figure 2 further links the observed corrosion morphology to the measured tensile properties of representative Q620 specimens. The nominal yield strength *f_y_*, nominal ultimate strength *f_u_*, and ultimate strain *ε_u_* are provided directly in each panel. Both strength parameters generally decreased with increasing mass loss, while *ε_u_* showed greater scatter but deteriorated markedly at high corrosion levels. Corrosion was approximately uniform for mass-loss ratios of 5–20%, whereas increasingly non-uniform damage was observed at higher levels [36].

A common conclusion of the available studies is that corrosion-induced ductility loss is usually more critical than strength loss. Tests on Q620 and Q690 steels showed reductions in elastic modulus, yield strength, ultimate strength and ultimate strain, but the degradation of ultimate strain and fracture morphology was more pronounced than the change in yield strain [36,37]. This suggests that corrosion assessment based only on residual yield strength may be insufficient for HSS structures. Indicators such as pit depth, surface roughness, corrosion-layer integrity and residual elongation should also be considered.

The literature also shows a methodological shift from average corrosion indices to local-damage descriptors. Mass loss and average corrosion depth remain useful for simple degradation models, but they cannot fully describe the stress concentration caused by pits. Recent studies therefore used maximum pit depth, surface roughness, Gumbel distribution models and thickness-dependent prediction models to describe localized corrosion more explicitly [37,38,39]. This development is valuable, because local defects are also the direct link between corrosion, fatigue crack initiation and member resistance degradation.

Environmental complexity remains a major limitation of current research. Most data were obtained from accelerated laboratory corrosion, while real structures experience coupled chloride attack, tidal wetting, flow, biological fouling and cathodic protection. Flowing seawater tests showed that flow velocity accelerated corrosion kinetics and promoted deeper pitting, while field exposure tests showed that dry–wet cycles and macrofouling increased hydrogen permeation and susceptibility to stress corrosion cracking [39,40]. Future corrosion models for HSS should therefore move from isolated mass loss correlations toward durability models with multiple parameters that include local pitting, plate thickness, environment and stress-assisted damage.

### 2.4. High-Temperature and Post-Fire Properties

Post-fire tests indicate that the residual material response of HSS is controlled by exposure temperature, cooling method, steel grade and forming process. For Q620, Q690 and UHSS, yield strength, ultimate strength and ductility changed more significantly than the elastic modulus after fire exposure, and predictive equations or constitutive models were commonly proposed for residual stress–strain curves [21,41,42,43,44,45]. Different cooling methods may result in distinct post-fire responses because of variations in cooling rate, microstructural evolution and material interaction [42,43,46]. Cold forming introduces additional variation in residual behaviour, as shown by tests on Q1100 UHSS and cold-formed HSS with nominal yield strengths of 700 and 900 MPa [44,47]. Some studies also indicate that post-fire assessment should extend beyond residual strength. Pre-tension during heating and cooling affected the residual strength of Q960 steel, while stress triaxiality and Lode angle influenced post-fire fracture strain of Q690 steel [48,49]. Post-fire fatigue and ultra-low-cycle fatigue studies show that fire exposure can alter fatigue resistance and may cause marked deterioration after severe heating [50,51]. Studies considering combined corrosion and fire exposure further indicate reduced fracture tolerance when corrosion pits act as local defects [52]. 

**Figure 3:**
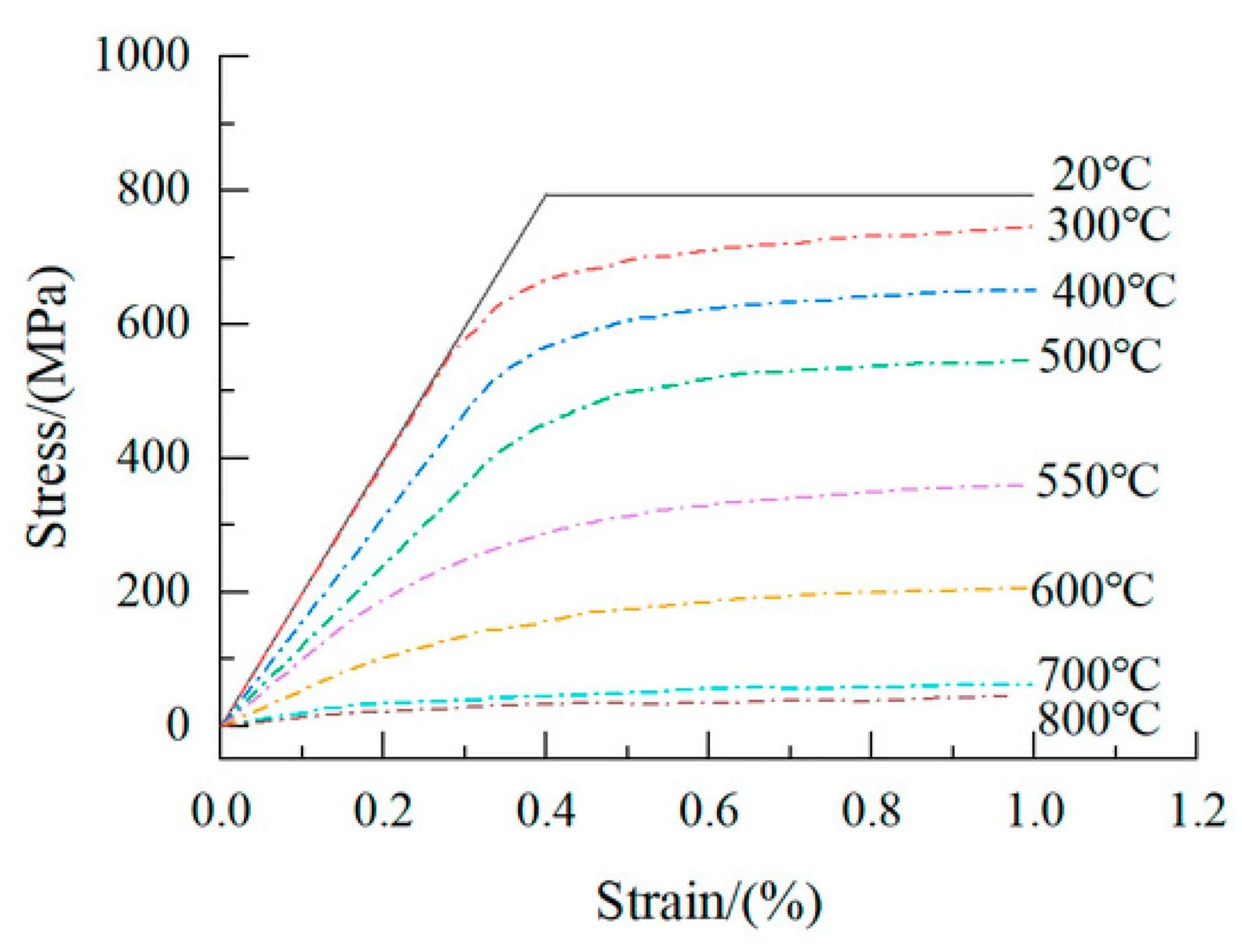
Degradation of stress–strain response of Q690 HSS with increasing elevated temperature. Adapted from the numerical study of Wang et al. [65].

Available elevated-temperature studies show that HSS material models should not be directly taken from mild-steel fire design provisions. For example, the Q690 stress–strain response in Figure 3 shows clear reductions in stiffness and strength as the temperature increases. Tests on BISPLATE 80, Q550, Q690, Q890, cold-formed 700/900 MPa HSS and Q960 steel showed that elastic modulus, yield strength, ultimate strength and ductility reduction factors are affected by steel grade, manufacturing route, test method and strain rate [53,54,55,56]. This issue becomes more pronounced for UHSS and cold-formed steels. Tests on Q1100 and Q1200 cold-formed UHSS sections showed nonlinear stress–strain curves without a clear yield plateau, significant strength reduction at elevated temperatures, and different responses between flat and corner regions [57,58]. In addition, studies on Q460, Q690D, Q890D and Q690 steels showed that fire-cooling-stage properties depend on both the current temperature and the prior peak temperature, so they cannot always be replaced by heating-stage properties at the same temperature [59,60,61,62]. For advanced fire simulation, reduction factors alone are insufficient. True stress–strain models developed for fracture simulation of Q550, Q690, Q890, Q960, S690 and S960 steels emphasize the importance of post-necking behaviour [63]. Creep rupture tests on Q550, Q690 and Q890 steels further show that time-dependent deformation and rupture should be considered when large deformation or failure is expected [64]. The representative studies on elevated-temperature and post-fire material properties are summarized in Table 3.

Overall, the reviewed studies used different definitions of yield strength, temperature ranges, strain rates, material locations and thermal histories. Heating-stage properties were measured at the current test temperature, whereas fire-cooling properties also depended on the prior peak temperature. Post-fire properties were measured after complete cooling and were further influenced by the cooling method. In addition, Q960 results showed strain-rate dependence, while Q1100 and Q1200 studies distinguished between flat and cold-formed corner material. Cross-study trends should therefore be interpreted within the corresponding material and test conditions, while broader unified relationships require further consistently designed experiments.

## 3. Structural Behaviour of High-Strength Steel Members

### 3.1. Residual Stresses

Residual stress is a key initial imperfection in HSS members, arising mainly from welding, cold forming, and cutting processes. It can influence yielding, buckling, and fatigue performance. Experimental evidence indicates that although the absolute magnitude of residual stresses in HSS members may be high, their ratio to yield strength is generally lower than that in conventional steel members, implying a reduced relative effect on structural resistance [18,66].

**Figure 4:**
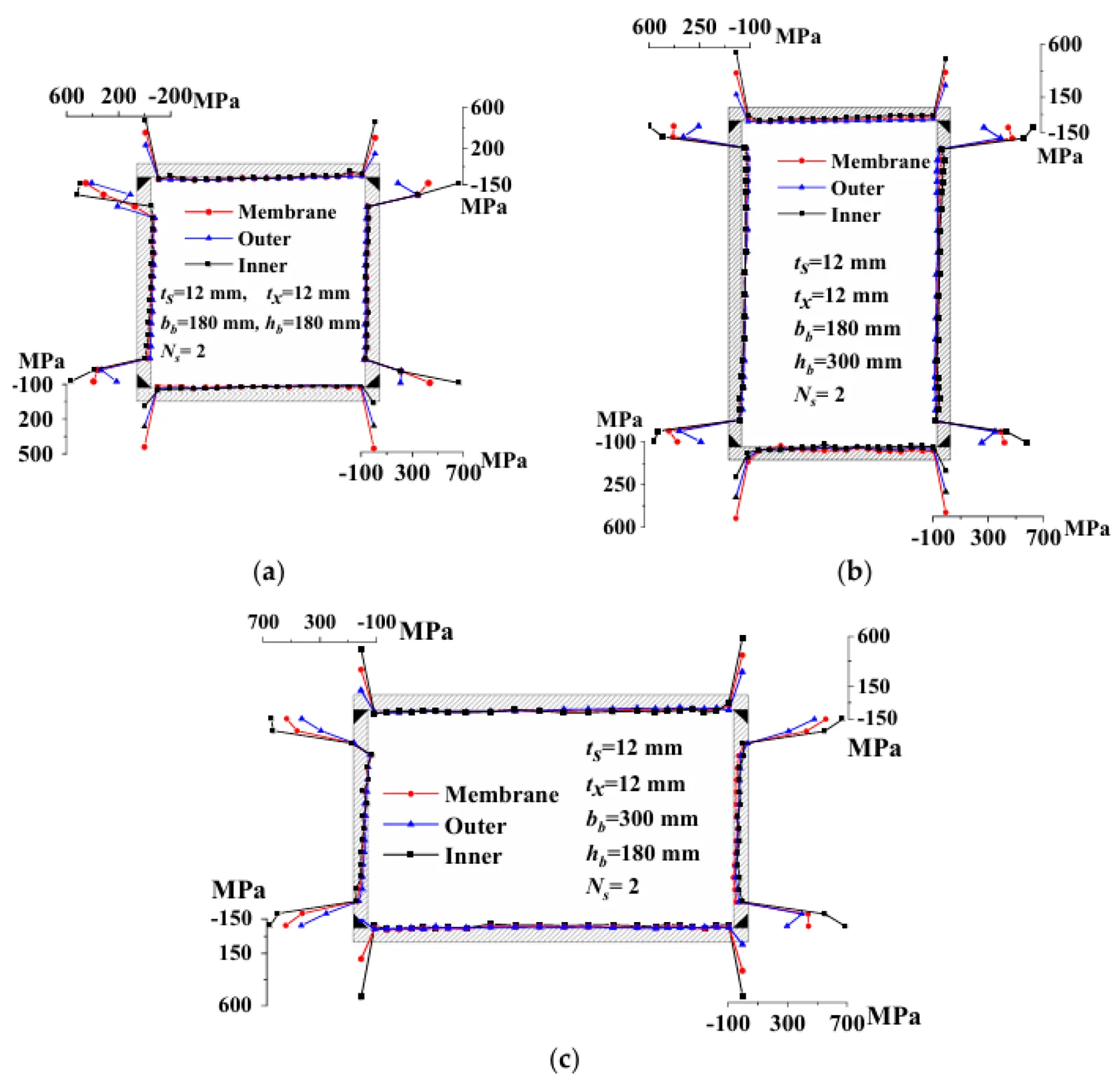
Measured residual stress distributions in welded HSS box sections with different cross-sectional dimensions: (**a**) square box section; (**b**) deep rectangular box section; (**c**) wide rectangular box section. Adapted from Wang et al. [74].

For welded built-up sections, residual stress distributions typically comprise tensile stresses concentrated near welds that are balanced by compressive stresses in adjacent plate regions. Tests on Q690, S690, and S960 welded sections confirmed this general pattern and showed that normalized compressive residual stresses tend to decrease with increasing steel grade. Existing studies have also demonstrated that residual stress distributions vary with section geometry and plate dimensions, leading to the development of section-specific predictive models for welded H-, box-, π-shaped, and cruciform sections [66,67,68]. The measured stress patterns in welded box sections further show that the distribution is sensitive to cross-sectional dimensions (Figure 4). Cold-formed hollow sections exhibit different characteristics because both membrane and bending residual stresses are generated during forming. Measurements on square hollow sections (SHS), rectangular hollow sections (RHS) and circular hollow sections (CHS) with strengths up to 1100 MPa showed that bending residual stresses can be significant and may promote early local yielding [18]. For cold-formed Q960 CHSs, residual stresses were found to have limited influence on stocky members but should be considered in the analysis of more slender sections [69].

Studies on welded circular tubes further highlighted the influence of manufacturing method. Significant longitudinal tensile residual stresses were observed near welds, and different stress patterns were identified for submerged-arc welded and high-frequency welded tubes [70]. Additional studies have shown that residual stresses may be substantially relaxed after fire exposure, while metallurgical effects such as phase transformation and heat-affected-zone softening can influence residual stress development in UHSS welded joints [71,72]. Numerical investigations also suggest that geometric imperfections may have a greater effect on buckling resistance than residual stresses in some HSS members, although residual stresses remain relevant in refined analyses [73].

Overall, residual stresses in HSS members are strongly dependent on the fabrication process, section type, and thermal history. Existing evidence suggests that their relative influence generally decreases with increasing steel strength, but accurate modelling remains necessary for welded members, slender sections, and structures subjected to fatigue or fire conditions.

### 3.2. Columns

Column members are among the most extensively studied HSS components. As shown in Table 4, research has focused on cross-sectional resistance, member buckling, cyclic behaviour, and residual performance after fire or environmental deterioration. For stub columns, local buckling is typically the governing failure mode. Studies on cold-formed SHS, RHS, CHS, and octagonal tubular sections highlighted the influence of plate slenderness, geometric imperfections, corner strengthening, and fabrication method on cross-sectional resistance and classification [75,76,77,78]. Studies on S960, Q1100 and Q1200 angle and channel sections showed that local buckling generally governed the resistance, and grade- and section-specific modifications to the examined design methods were proposed [79,80,81]. For built-up and lipped channel sections, local–distortional and local–distortional–global interactions were significant, motivating modified design approaches based on the direct strength method (DSM) [82,83].

For slender columns, global buckling generally governs when local buckling is restrained. Experimental and numerical studies on Q690, S690, and S960 welded box, H- and I-sections confirmed the predominance of flexural buckling in compact members [84,85,86,87]. Residual stresses have a reduced relative influence in HSS columns, while the reported normalized buckling resistance remains sensitive to the adopted column curve and section family [88,89]. Open sections may exhibit flexural–torsional buckling, orientation-dependent flexural buckling, or local–flexural interaction, depending on the cross-sectional shape and member slenderness [90,91,92]. Under cyclic loading, welded HSS columns generally exhibit stable hysteretic behaviour, with ductility governed mainly by width-to-thickness ratio, axial load level, and member slenderness [93,94].

Artificial grooving corrosion in Q1100 channel stub columns further reduced stiffness and resistance and localized buckling near the weakened region [95]. At elevated temperatures, HSS columns retain similar buckling modes but undergo marked reductions in strength and stiffness governed by material degradation, imperfections, local buckling and slenderness [96,97,98]. Post-fire studies on S700, Q550, Q690 and Q960 columns showed that residual resistance depends on peak temperature, section geometry, eccentricity, restraint and residual deformation [99,100,101,102]. The accompanying numerical study indicated similar trends for Q550, Q690 and Q890 columns, but the exact factors should not be directly extended to other section types because of differences in thermal, restraint, geometric and post-fire material conditions [101].

**Figure 5:**
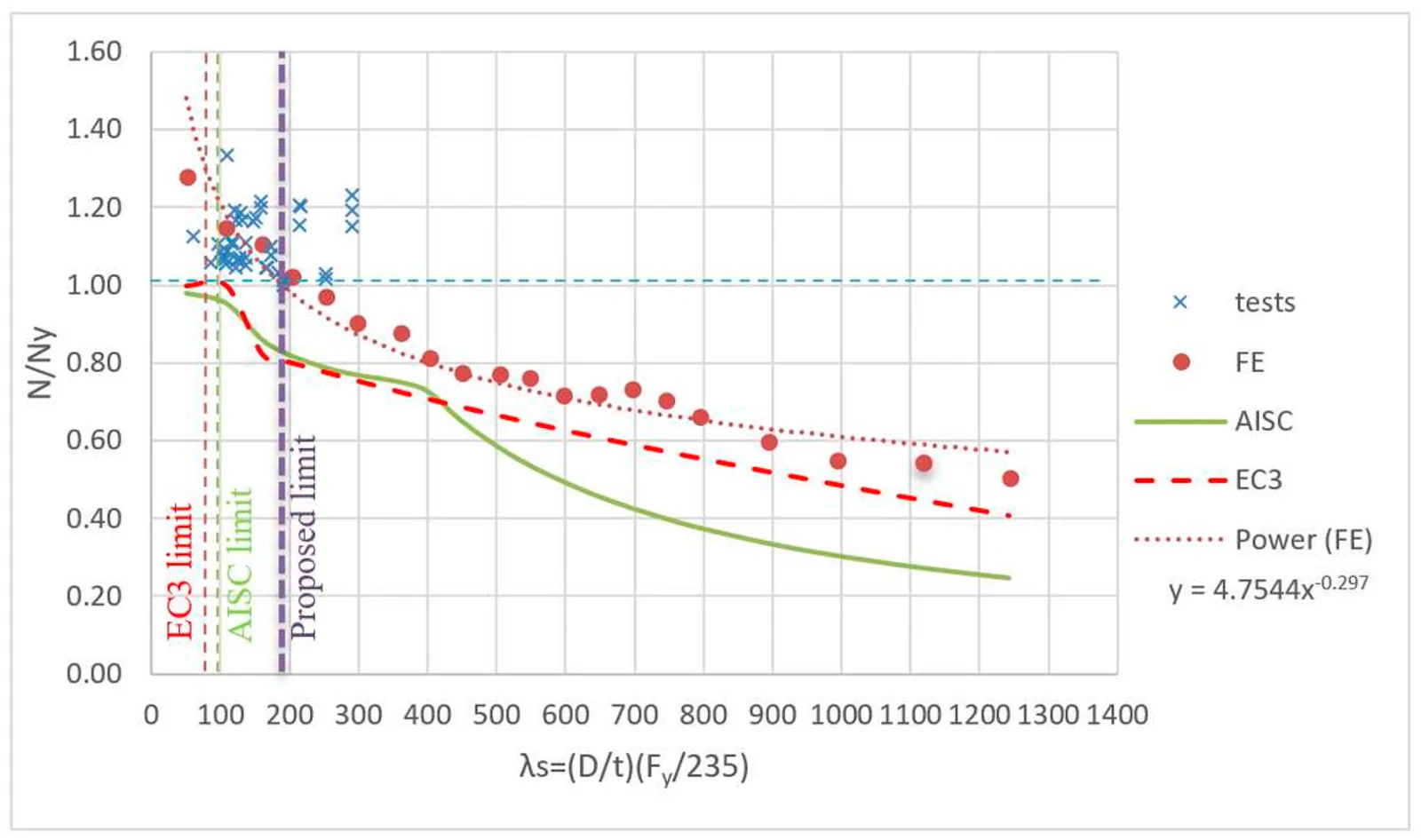
Normalized axial compression resistance of S1100 UHSS CHS stub columns versus cross-section slenderness, compared with experimental, finite element, code, and proposed predictions. Adapted from Shaker et al. [105].

The continuous strength method (CSM) and machine-learning methods have been extended to HSS tubular stub columns and I-section columns, respectively, and were validated against the investigated HSS datasets [103,104]. Figure 5 compares the normalized axial compression resistance of S1100 UHSS columns with existing and proposed design predictions. Across the available studies, local buckling generally governs stocky sections, whereas global flexural buckling governs slender members when local instability is restrained. However, the reported accuracy of existing column curves varies among welded, cold-formed, open and tubular sections. These differences are mainly associated with cross-section slenderness, fabrication route, residual stresses, geometric imperfections and local–global interaction. Modified column curves, the continuous strength method, the direct strength method and data-driven models should therefore be applied only within the steel grades, section families and slenderness ranges covered by their validation datasets.

**Table 4:** Summary of studies on HSS column members.

References	Steel Grade	Section Type	Loading Condition	Key Finding
Ma et al. [75]	S700, S900, and S1100	SHS, RHS, CHS	Axial compression	Local buckling and plate slenderness governed resistance; numerical modelling captured tests and supported design assessment.
Ma et al. [76]	S700, S900, and S1100	SHS, RHS, CHS	Axial compression; numerical parametric study	Existing cold-formed tubular-section design rules were assessed, and modified approaches were proposed for HSS tubes.
Chan et al. [77]	HSS (greater than 700 MPa)	RHS, CHS	Axial compression	Current cross-section slenderness limits were assessed, and revised parameters were proposed to improve design efficiency.
Fang et al. [78]	S690	Octagonal tubular sections	Axial compression	Fabrication route affected material heterogeneity, imperfections, local buckling response, and design resistance.
Ban et al. [84]	S960	Welded I- and box-sections	Axial compression	Specimens failed by overall flexural buckling, and higher column curves were suggested for S960 welded columns.
Li et al. [85]	Q690	Welded box and H sections	Axial compression	Tests showed global buckling of compact sections and were used to assess existing column curves.
Li et al. [88]	Q690	Welded box and H sections	Axial compression; parametric study	Refined plastic-hinge analysis supported design recommendations for Q690 welded columns.
Ma et al. [86]	S690	Welded H sections	Axial compression	All columns failed by minor-axis flexural buckling; EN and GB rules were conservative, while AISC predictions were closer.
Ferreira Filho et al. [87]	S690	Welded I sections	Axial compression; compression and bending	Test and numerical data were used to assess current flexural-buckling design methods for S690 welded I-sections.
Fang et al. [89]	S700, S900 and S1100	Cold-formed SHS, RHS, CHS	Axial compression; numerical parametric study	Higher steel grade increased normalized column strength and reduced sensitivity to geometric imperfections.
Wang et al. [79]	S960	Angle and channel sections	Axial compression	Local buckling dominated, and existing rules were not directly applicable without modification for S960 open sections.
Zhang et al. [90]	S690	Press-braked angle sections	Axial compression	Flexural-torsional buckling was critical, and DSM-based methods provided more rational resistance predictions.
Wang et al. [91]	S960	Press-braked channel sections	Axial compression	Minor-axis flexural buckling governed, while the buckling orientation significantly affected the column resistance.
Sun et al. [92]	S690	Welded I sections	Axial compression	Local–flexural interactive buckling was observed, with marked variation among the examined code predictions.
Xue et al. [80]	Q1200	Angle and channel sections	Axial compression; tests and numerical analysis	Cold-forming reduced ductility, and revised design approaches were proposed for UHSS open-section stub columns.
Hua et al. [81]	Q1100	Angle and channel sections	Axial compression; tests and numerical analysis	Local buckling governed, and the examined European, American and Australian/New Zealand design approaches required modification for Q1100 sections.
Cui et al. [82]	G550	Built-up OI and CB sections	Axial compression	Local, distortional, and global interactions affected strength, and modified DSM equations improved predictions.
Wang et al. [83]	Q460	Lipped channel sections	Axial compression	Slenderness and flange width governed global buckling, while aspect ratio, lip width, and thickness affected interactive buckling.
Chen et al. [93]	Q690D	Welded H sections	Constant axial load and cyclic lateral loading	Stable hysteretic behaviour was observed before local buckling, and a trilinear moment-curvature hysteretic model was proposed.
Yang et al. [94]	Q690	Welded box sections with slender webs	Low-cycle lateral loading	Plastic local buckling controlled failure; width-to-thickness ratio, axial load ratio, and slenderness reduced ductility.
Chen and Young [96]	690 MPa HSS	Box and I sections	Elevated temperatures; numerical study	Reduced material properties and initial imperfections were incorporated to assess design rules for HSS columns in fire.
Wang et al. [97]	Q460; Q235 comparison	Welded H sections	Axial compression at room and elevated temperatures	Local buckling modes were similar at room and elevated temperatures, but Q460 capacity degraded rapidly under fire.
Fang and Chan [98]	S355, S450, S700, and S900	Cold-formed SHS and RHS	Elevated temperatures up to 800 °C; numerical study	Existing elevated-temperature methods were assessed numerically for SHS/RHS columns, and modified expressions were proposed.
Zhong et al. [99]	S700	SHS tubular sections	Post-fire eccentric compression; 400–1000 °C	Residual resistance was stable after 400–600 °C but decreased markedly after higher temperatures.
Li et al. [101]	Q550	H sections	Heating-cooling cycle and post-fire compression	Restraint ratio was 0.37; load ratios were 0.27 and 0.35; residual capacity factors were 0.54 and 0.56 and stiffness factors were 0.32 and 0.33; residual capacity and stiffness were reduced to about 55% and 32% of initial values.
Song et al. [102]	Q690	H sections	Whole heating-cooling cycle; post-fire loading	Post-fire reusability depended on restraint and failure mode; yielding-dominated columns retained higher capacity than buckling-dominated columns.
Xue et al. [100]	Q960	Cold-formed channel sections	Post-fire numerical study; 600–900 °C	The exposure temperature and cross-sectional geometry governed the residual resistance, and modified design equations were proposed.
Xue et al. [95]	Q1100	Cold-formed channel sections	Grooving corrosion; axial compression	Artificial grooving corrosion reduced the initial stiffness and resistance and promoted local buckling near the weakened region.

### 3.3. Beams

**Figure 6:**
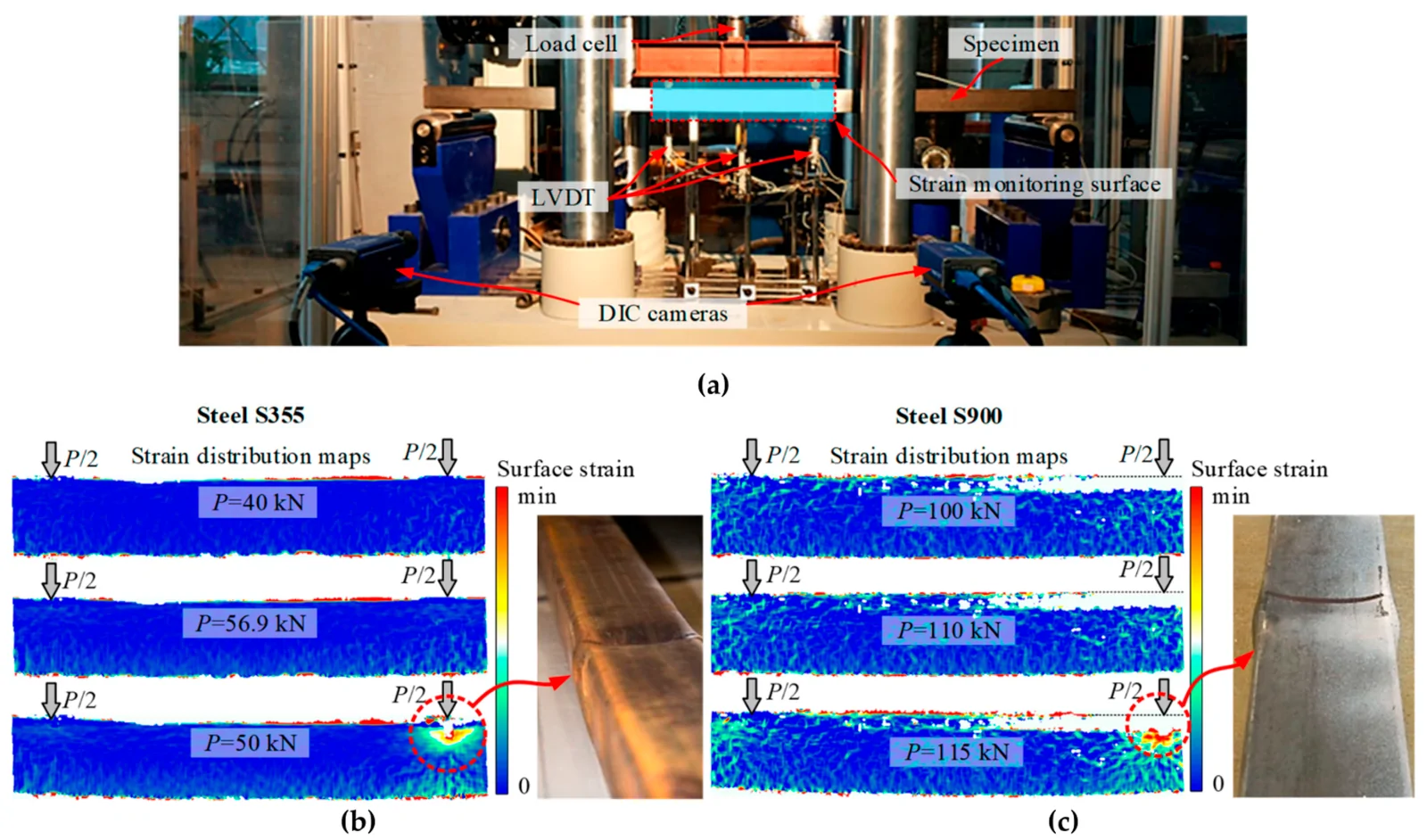
Comparative four-point bending behaviour of normal-strength steel and HSS square hollow-section beams: (**a**) test setup; (**b**) normal-strength steel beam; (**c**) HSS beam. Adapted from Misiūnaitė et al. [106].

Research on HSS beam members has mainly focused on flexural behaviour, cross-section classification, local and global stability, and elevated-temperature performance (Table 5). Existing investigations covering tubular sections, welded I-sections, and cold-formed or press-braked open sections consistently demonstrate that HSS beams can achieve high flexural resistance and make efficient use of material strength. A comparison between normal-strength and high-strength square hollow section beams is presented in Figure 6. However, the increase in material strength is not always fully translated into a proportional increase in member resistance [106]. The reported prediction accuracy of beam design methods varies with section slenderness, steel grade and fabrication route [107,108,109,110]. The direct applicability of cross-section classification limits and flexural resistance formulations developed mainly for conventional steels therefore requires case-specific verification for slender, press-braked and UHSS beams. Stability-related failure modes also require particular attention. For open thin-walled sections, local buckling, distortional buckling, and their interactions become increasingly significant as steel strength increases, while lateral–torsional buckling remains a key consideration for beams [111,112]. These findings suggest that future design approaches may need to place greater emphasis on stability effects rather than relying solely on strength-based considerations.

Research on HSS beams under fire conditions remains limited. For cold-formed 700/900 MPa SHS and RHS beams at elevated temperatures, Li and Young [113] assessed existing fire-design provisions and proposed modified expressions. The supporting experimental database remains limited. For tubular beams, modified DSM and CSM rules improved flexural resistance predictions for HSS SHS, RHS, CHS, and elliptical hollow sections (EHS) [114,115]. Plate slenderness and local buckling are the main limits on HSS beam resistance and rotation capacity. The reported prediction variability increases for slender, press-braked and cold-formed sections, particularly when local–distortional interaction occurs. These differences reflect section form, bending axis, fabrication-induced material variation, boundary conditions and the treatment of strain hardening.

### 3.4. Beam-Columns

Beam-columns are governed by compression–bending interaction, member instability, local buckling, and cyclic deterioration in seismic cases. Tests and numerical studies on Q460C box sections, Q690 welded H-sections, and cold-formed SHS and RHS members with nominal yield strengths of 700 and 900 MPa showed that local and global buckling, section slenderness, and loading eccentricity control the resistance [116,117,118,119]. For 51 short cold-formed SHS and RHS beam-columns, the mean test-to-prediction ratios were 1.13, 1.21 and 1.16 for ANSI/AISC 360-10, AS 4100:1998 and EN 1993-1-1:2005, respectively, with corresponding coefficients of variation (COVs) of 0.081, 0.075 and 0.067 [119]. The reliability indices were 2.94, 3.01 and 2.51, respectively. Studies on S700 built-up sections and S960 and Q1100 welded I-sections reported different prediction trends among the examined European, American, Australian and Chinese interaction methods [120,121,122]. The differences were associated with member slenderness, section family and the resistance end points adopted in the interaction equations.

Under cyclic loading, Q460 and Q690 welded H- and box-section beam-columns can show stable hysteretic behaviour when compact sections are used, but ductility and energy dissipation are strongly affected by plate slenderness, axial load ratio, bending axis, cyclic softening, and ductile damage [123,124,125,126,127]. Post-fire Q960 beam-columns showed that residual resistance depends on exposure temperature, eccentricity, slenderness, and degraded material properties, while Q690 frame studies also highlighted the importance of restraint effects at elevated temperatures [128,129].

Representative studies on HSS beam-columns are summarized in Table 5. Compression–bending interaction, member slenderness and local buckling are consistently identified as the principal controls on HSS beam-column behaviour. However, the reported bias of existing interaction rules is not uniform. This difference is mainly related to member slenderness, section family, bending axis and the resistance end points adopted in the interaction equations. The reported model statistics should therefore not be extended beyond the tested geometries and loading conditions.

### 3.5. Plate Girders and Web Crippling

**Figure 7:**
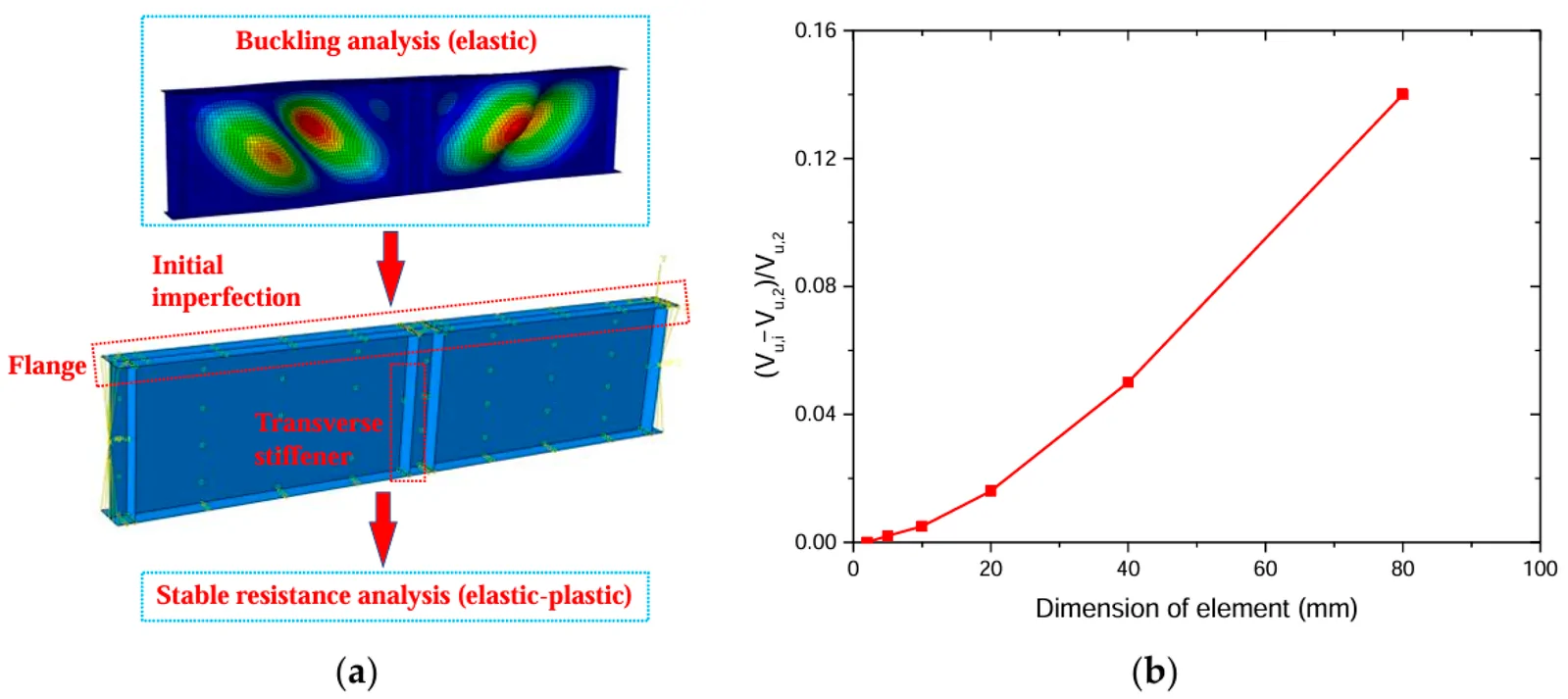
Numerical model and mesh convergence study for post-fire Q690 HSS plate girders: (**a**) finite element model; and (**b**) convergence study. Adapted from Liu et al. [143].

Plate girders and members susceptible to web failure have been studied mainly in relation to shear post-buckling, patch loading, web crippling, and fire-related resistance. For Q550 and Q690 HSS plate girders, numerical studies showed that strain hardening affects post-buckling shear capacity, and that design equations developed for mild steel may not directly apply to HSS plate girders [130]. Patch-loading behaviour has also been examined. Numerical studies on Q460, Q550, Q690, and Q960 plate girders showed that steel grade, web slenderness, loading length, and web aspect ratio strongly affect local bearing resistance [131]. Fire-related studies further showed that elevated temperature and post-fire exposure reduce the resistance of Q690 plate girders, especially through material degradation and reduced postbuckling reserve, and new prediction methods were therefore proposed for serviceability and ultimate limit states [132,133,134]. A representative numerical modelling scheme for post-fire Q690 HSS plate girders is shown in Figure 7.

For web crippling, tests and numerical studies on cold-formed HSS SHS/RHS and S690/S960 channel sections showed that web slenderness, bearing length, loading condition, and corner material properties govern local bearing resistance [135,136,137]. For HSS tubular sections, the reported accuracy of the examined design methods varied under combined bending–bearing and post-fire web-crippling conditions [138,139]. Modified DSM rules were proposed for HSS tubular sections under the four standard loading conditions [140]. Related studies on high-strength stainless steel hollow sections also showed that conventional web crippling and combined bending–web crippling rules may require recalibration for nonlinear metallic materials [141,142].

In general, the resistance of HSS plate girders and members prone to web crippling is governed by factors such as web slenderness, load length, stiffener arrangement, and post-buckling behaviour. The reported accuracy of design methods varies across shear, patch loading, and web-crippling scenarios due to the distinct stress fields and boundary conditions associated with each failure mode. Exposure to fire introduces additional variability by causing material degradation and altering post-buckling progression. Since most proposed equations are derived from specific families of cold-formed sections, their application should be confined to the reported ranges of geometry, loading, and temperature conditions.

## 4. Connections and Joints

### 4.1. Welded Connections

Welded connections are critical in HSS structures because welding creates significant material heterogeneity in the HAZ. Existing studies consistently show that HAZ softening is one of the main factors governing the behaviour of HSS butt-welded joints. Joint resistance is influenced by the degree and width of softening, weld-metal matching, and the constraint provided by adjacent stronger material [144,145,146,147]. Although local softening does not always lead to a substantial reduction in ultimate strength, it can significantly affect deformation capacity and should be considered in design [148]. 

**Figure 8:**
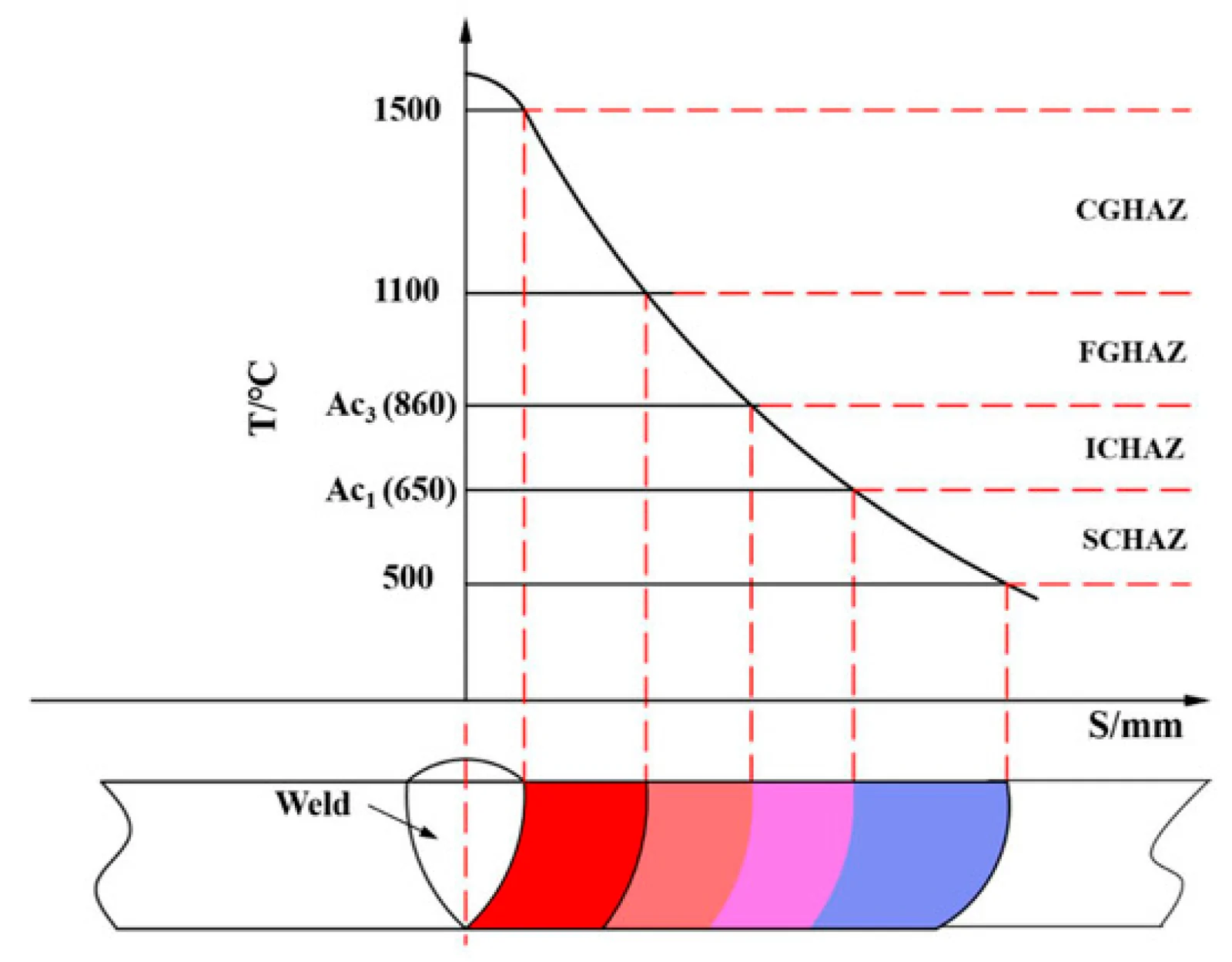
Schematic division of the heat-affected zone in welded Q690 high-strength bridge steel (CGHAZ = coarse-grained heat-affected zone; FGHAZ = fine-grained heat-affected zone; ICHAZ = intercritical heat-affected zone; SCHAZ = subcritical heat-affected zone). Adapted from Zhang et al. [156].

Material characterization and thermal simulation studies further indicate that different HAZ subregions may exhibit either softening or hardening depending on the welding thermal cycle and resulting microstructural transformations. These findings provide a basis for developing more realistic material and fracture models for welded HSS connections [16,148]. The typical HAZ regions in welded Q690 bridge steel are illustrated schematically in Figure 8. For fillet and cruciform welded joints, heat input, welding procedures, and local restraint have been identified as important parameters affecting post-weld mechanical properties. Experimental and data-driven studies have shown that strength variations caused by welding can be spatially non-uniform and are relevant to the assessment of joint performance [149,150].

Fatigue and corrosion effects have also received considerable attention. Fatigue resistance is sensitive to joint geometry, weld details, and corrosion damage, while corrosion can markedly reduce fatigue resistance and residual strength [151,152,153,154,155]. Experimental testing, three-dimensional surface characterization and data-driven approaches have been used to evaluate the durability of welded HSS connections under aggressive environments. 

The HAZ heterogeneity, strength matching, heat input and local ductility are consistently identified as important factors in welded HSS connections. However, the magnitude and location of softening vary with steel grade, delivery condition, welding procedure and plate thickness, which explains the different reported fracture locations and strength reductions. Existing constitutive and resistance models are therefore dependent on the investigated steel and welding process. Since most evidence remains at coupon or detail scale, transfer to full-scale connections requires further validation.

### 4.2. Bolted Connections

Bolted connections are widely used in HSS structures because of their ease of fabrication and installation. For bearing-type connections, tests on Q550D, Q690D, and Q890D specimens showed that end distance, edge distance, and bolt arrangement significantly influence failure mode and resistance. Tear-out and splitting were the dominant failure modes, with splitting generally resulting in lower strength [157,158]. Numerical studies further indicated that shear fracture around bolt holes is governed by strain localization and tensile stress concentration, and that conventional bolt-hole elongation limits may overestimate deformation capacity [159,160].

Studies on net-section fracture showed that Q960 and other HSS bolted connections can still achieve adequate deformation capacity despite reduced material ductility [161]. Fire and post-fire investigations indicated that material degradation affects tensile resistance, while net-section efficiency is less sensitive to temperature exposure [162,163]. Block tearing has been investigated for S700 connections under ambient and post-fire conditions [164,165]. The cited studies compared existing block-tearing provisions with test and numerical results and attributed the reported prediction bias mainly to the treatment of the shear failure plane.

**Figure 9:**
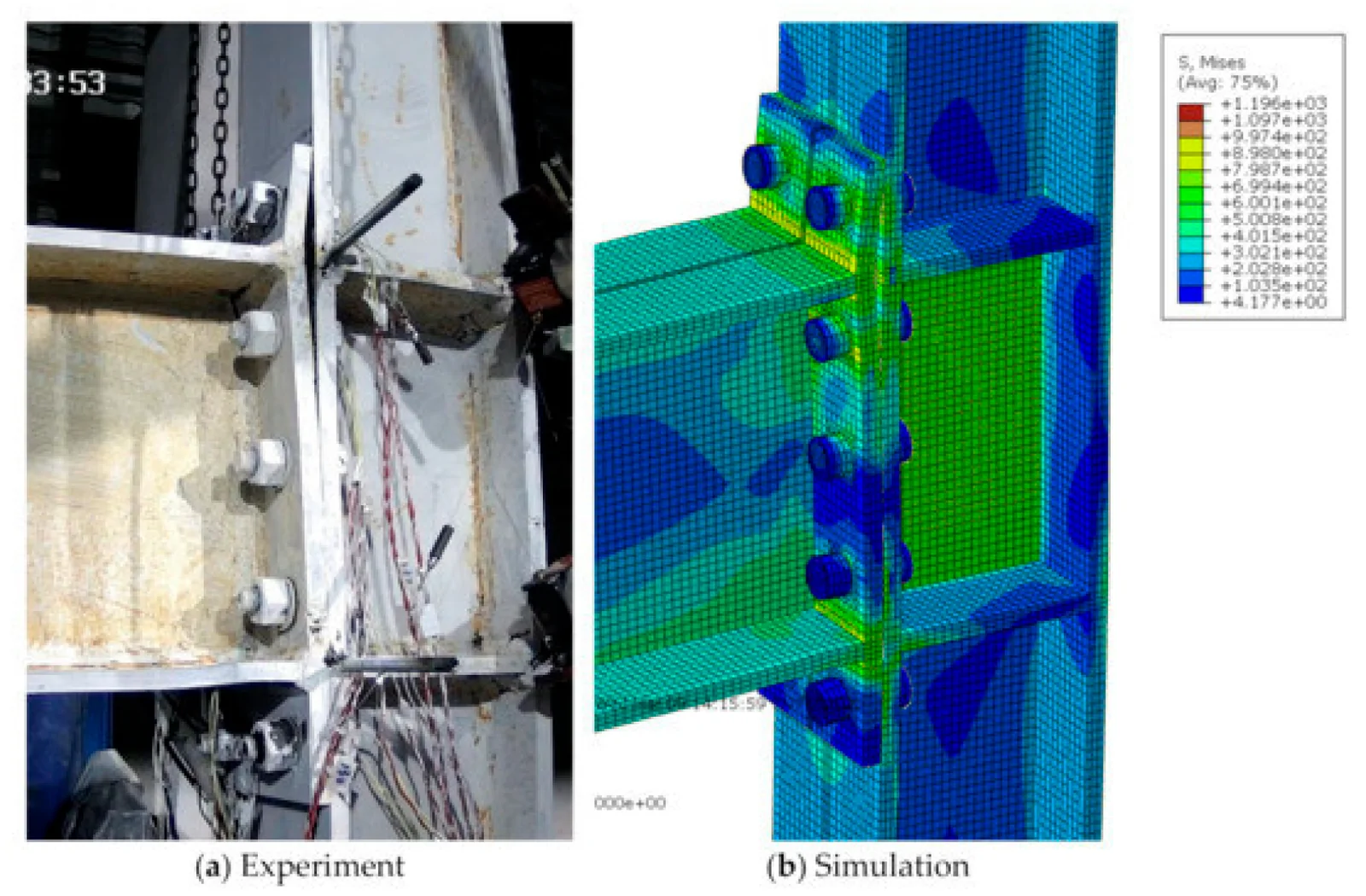
Experimental and numerical comparison of HSS extended end-plate connection under cyclic loading. Adapted from Lin et al. [167].

Additional studies examined slip-critical and end-plate connections. A slip factor of 0.40 was recommended for shot-blasted surfaces of Q550, Q690 and Q890 steels, while the beneficial effect of rusting was considered unreliable for design [166]. Tests and numerical analyses of extended end-plate connections highlighted the influence of end-plate deformation, contact behaviour and cyclic loading on connection performance (Figure 9) [167]. For bolted HSS connections, end distance, edge distance, bolt arrangement and the governing failure mode are widely confirmed as the main variables. The reported conservatism of existing provisions differs among bearing, net-section fracture, block tearing and slip-critical behaviour. The recommended slip factor of 0.40 should not be generalized to other surface treatments. Design conclusions should be stated together with the steel grade, connection geometry, failure mode and surface condition.

### 4.3. Tubular Joints and Special Connections

Tubular joints are critical components of HSS tubular structures because local failure at the brace–chord intersection may govern the resistance of trusses, frames, bridges, and offshore structures. Studies on CHS X-joints with steel grades up to S1100 showed that the strength equations in Eurocode and those proposed by CIDECT (Comité International pour le Développement et l’Étude de la Construction Tubulaire) may become unconservative as steel grade increases, especially for joints governed by chord face plastification [168]. 

Tests and numerical simulations further showed that joint behaviour depends on steel grade, brace-to-chord geometry, chord slenderness, HAZ properties, and failure mode. For the examined Q890 and Q960 joints, welding with low heat input was adopted. HAZ softening was minor in the Q960 tests, while numerical analyses indicated that local HAZ strength reduction could decrease the resistance of fabricated RHS joints. However, the existing Eurocode and CIDECT equations did not provide consistently reliable predictions. They could be unconservative for chord-face plastification but conservative for chord side wall failure or combined failure modes [17,169]. Cold-formed S900 and S960 tubular T- and X-joint tests further showed that EC3/CIDECT prediction accuracy depended on joint configuration, support condition, geometry and failure mode [170,171,172]. Limited studies have addressed composite and spatial connections. Concrete-filled HSS tubular X-joints exhibited ductile load–deformation responses, and the CIDECT equations were evaluated against the reported tests [173]. Q690 bolted joints connecting H-section members to hollow spheres were also proposed for assembled suspend-dome structures under combined compression and bending [174].

Overall, HSS tubular and spatial joints cannot be evaluated by simply scaling normal-strength steel equations according to yield strength. Failure mode, joint geometry, HAZ behaviour, chord slenderness, composite action, and loading eccentricity should be considered together. Further full-scale tests, cyclic and fatigue studies, and design formulations specifically calibrated for HSS and UHSS joints are required. The key geometric parameters and reported design comparisons for selected slip-critical and tubular connections are summarized in Table 6. 

## 5. Conclusions

This review summarized the material behaviour, degradation mechanisms and component performance of structural HSS. The main conclusions are as follows.

1. Constitutive behaviour. Higher yield-to-tensile strength ratios, shorter or absent yield plateaus and reduced strain-hardening capacity are commonly reported for HSS. Cyclic softening, stress-state dependence, strain rate and temperature should be considered when relevant. Model parameters remain dependent on steel grade, product form and manufacturing route.
2. Fatigue and fracture. Material and detail tests confirm that loading history, stress state, corrosion pits and surface roughness affect cyclic degradation, crack initiation and fracture. However, most models were calibrated for specific grades, specimens and loading paths. Their applicability to variable-amplitude, multiaxial and component-level fatigue requires further validation.
3. Corrosion and fire. Corrosion of Q620 and Q690 steels showed that local pitting and ductility loss cannot be represented by average section loss alone. Elevated-temperature and post-fire studies further showed that strength and ductility depend on steel grade, forming route, peak temperature and cooling method. Heating-stage properties should not be applied directly to cooling and post-fire conditions without verification.
4. Structural members. Local buckling governs many stocky and thin-walled members, whereas global or interactive buckling becomes important for slender columns and open sections. Reported design-model accuracy varies with steel grade, section family and failure mode, with greater variability reported for UHSS open sections, cold-formed products and members involving local–distortional interaction. Evidence for beams, plate girders and web crippling remains less extensive than that for columns.
5. Connections. Welded-joint behaviour is strongly influenced by heat-affected-zone softening, strength matching, heat input and local ductility. For bolted connections, design accuracy depends on the governing failure mode and surface condition. Tubular-joint predictions are also failure-mode dependent, conclusions for chord-face plastification should not be extended directly to chord-sidewall or combined failures.

Further studies should focus on consistent material characterization across steel grades and product forms, coupled corrosion–fatigue–fire effects, broader experimental validation of members and connections, and design models with clearly defined applicability limits. Particular attention should be given to less extensively studied beams, plate girders, joints and structural systems, as well as to the transferability of numerical and data-driven models beyond their calibration ranges.

## Figures and Tables

**Table 1:** Representative HSS grades, designation systems and typical products.

Country/Region and Designation System	Representative Grades	Strength Designation	Typical Delivery Routes and Products
China: GB/T Q-series	Q460, Q550, Q620, Q690, Q890 and Q960	The number indicates the nominal yield strength in MPa.	Thermomechanically processed or quenched-and-tempered plates, welded and cold-formed sections.
Europe: EN S-series	S460, S500, S550, S690, S700, S890 and S960	The number indicates the nominal yield strength in MPa, subject to product thickness.	Thermomechanically rolled or quenched-and-tempered plates, welded sections and hollow sections.
United States: ASTM specifications	ASTM A709 HPS 70W and HPS 100W	70 and 100 denote yield strength in ksi for HPS grades.	Quenched and tempered structural plates, mainly used in buildings and bridges.

**Table 2:** Summary of studies on cyclic, fatigue, fracture and corrosion behaviour of HSS.

References	Steel Grade	Research Topic	Key Finding
Wang et al. [22]	Q460C	Cyclic behaviour and constitutive modelling	Hysteretic response, Bauschinger effect and simplified kinematic hardening model.
Wang et al. [23]	Q690D	Cyclic response and hysteretic modelling	Cyclic softening and combined isotropic–kinematic hardening hysteretic model.
Javidan et al. [24]	Grade 800/1200	Low-cycle damage	Cyclic softening, retained properties, ductility and energy dissipation of HSS/UHSS materials.
Zhang et al. [25]	Q1100	Hysteretic behaviour	Nonlinear response, cyclic degradation and comparison of constitutive models.
He et al. [26]	Q690	Stress-state-dependent cyclic plasticity	Limitations of conventional stress-state parameters under cyclic loading and a backstress-related parameter.
Wang et al. [27]	Q550, Q690 and Q890	Ductile fracture modelling	Fracture strain affected by both stress triaxiality and Lode angle; comparison of fracture models.
Shang et al. [28]	Q960	Stress-state-dependent plasticity and fracture	Plasticity, damage evolution and Hosford-Coulomb-type fracture modelling.
Xue et al. [29]	Q690	Residual monotonic properties after pre-fatigue	Effects of accumulated fatigue damage on residual strength, ductility and stress–strain curves.
Li et al. [30]	Q690E	Fatigue life prediction	Use of ultrasonic fatigue damage index for fatigue life and residual life prediction.
Zhu et al. [34]	EQ56	Multiaxial low-cycle fatigue	Effects of multiaxial strain ratio on cyclic response, fracture morphology and fatigue-life modelling.
Guo et al. [31]	Q690D	Corrosion fatigue	Stress-life curves after corrosion; fatigue limit decreased after corrosion damage.
Yue et al. [35]	Q690E, Q690qENH	Corrosion and high-cycle fatigue	Corrosion morphology, corrosion products and fatigue-life degradation of ordinary and weathering HSS.
Liu and Zong [32]	Q690D	Corrosion-induced roughness and cyclic behaviour	Influence of corrosion roughness on low-cycle fatigue life and hysteretic behaviour.
Guan et al. [33]	Q690D	Fatigue crack growth after corrosion	Effects of corrosion pits on fatigue crack growth rate and crack-growth threshold.
Xue et al. [36]	Q620	Mechanical properties after corrosion	Relationships between corrosion degree and strength or ductility degradation.
Wei et al. [37]	Q690	Mechanical properties after corrosion	Thickness effect, mechanical degradation and constitutive modelling of corroded Q690 steel.
Wei et al. [38]	Q355, Q690	Coastal atmospheric corrosion	Comparison of corrosion evolution of ordinary steel and Q690 HSS; time-dependent corrosion model.
Dan and Bao [39]	Q690	Flowing seawater corrosion	Effects of seawater flow on electrochemical corrosion, pitting morphology and tensile degradation.
Cai et al. [40]	AISI 4135	Dry-wet cycling and stress corrosion cracking	Hydrogen permeation and stress-corrosion-cracking susceptibility in marine exposure.

**Table 3:** Summary of studies on high-temperature and post-fire material properties of HSS.

References	Steel Grade	Research Topic	Key Finding
Chen et al. [53]	BISPLATE 80	Material properties at elevated temperatures	High-temperature strength, elastic modulus and thermal elongation of HSS compared with mild steel.
Li and Young [55]	Cold-formed 700/900 MPa HSS	Material properties at elevated temperatures	Reduction factors and design curves for cold-formed HSS at elevated temperatures.
Huang et al. [54]	Q550, Q690 and Q890	Material properties at elevated temperatures	Effects of steel grade on high-temperature reduction factors; limitations of current code models.
Sun et al. [56]	Q960	Strain-rate effect at elevated temperatures	Coupled influence of temperature and tensile strain rate on Q960 mechanical properties.
Shi et al. [57]	Q1100	Low- and elevated-temperature properties	Temperature-dependent stress–strain curves and constitutive model for cold-formed Q1100 UHSS sections.
Luo et al. [58]	Q1200	Low- and elevated-temperature properties	Temperature-dependent mechanical properties and constitutive model for cold-formed Q1200 UHSS sections.
Wang et al. [62]	Q690	Fire-cooling-stage properties	Cooling-stage influence coefficients and a model for heating, cooling and post-fire stages.
Jiang et al. [60]	Q460	Fire-cooling-stage properties	Cooling-stage properties governed by both current temperature and prior peak temperature.
Wang et al. [59]	Q690D	Fire-cooling-stage properties	Comparison between heating and cooling stages and discussion of microstructural effects.
Wang et al. [61]	Q890D	Entire-fire-process properties	Reduction factors and cooling-stage influence coefficients for the entire fire process.
Cai et al. [63]	Q550, Q690, Q890, Q960, S690, and S960	True stress–strain model at elevated temperatures	True stress–strain models for elastic, hardening and post-necking regions; S690/S960 included through available high-temperature datasets.
Yan et al. [64]	Q550, Q690 and Q890	Creep deformation and rupture	Creep deformation, creep rupture and temperature-dependent damage mechanisms.
Li et al. [41]	Q690	Post-fire mechanical properties	Post-fire yield strength, ultimate strength, elastic modulus, elongation and predictive equations.
Li and Young [47]	Cold-formed 700/900 MPa HSS	Post-fire mechanical properties	Predictive curves for residual mechanical properties of cold-formed HSS after fire exposure.
Zhou et al. [42]	Q620	Post-fire mechanical properties	Effects of cooling method on residual mechanical properties and necking behaviour.
Xue et al. [43]	Q960	Post-fire mechanical properties	Post-fire stress–strain curves, microstructure and predictive models for Q960 UHSS.
Wang et al. [48]	Q960	Post-fire properties under pre-tension	Influence of sustained tensile stress during fire exposure on residual strength and elastic modulus.
Hua et al. [44]	Q1100	Post-fire mechanical behaviour	Microstructure, residual strength, ductility and constitutive model after elevated-temperature exposure.
Wang et al. [21]	Q1100	Post-fire deterioration	Effects of cold forming and prior elevated-temperature exposure on strength, ductility and fracture behaviour.
Xiao et al. [45]	Q1200	Post-fire properties after exposure to 20–1000 °C	Residual properties depended strongly on temperature and cooling method; predictive equations were proposed.
An et al. [50]	Q690	Post-fire fatigue properties	Fatigue life, fracture surface, metallography and fatigue-life prediction model after fire exposure.
Hua et al. [51]	Q690	Post-fire ultra-low-cycle fatigue	Effects of cooling method on ultra-low-cycle fatigue behaviour, cyclic curves and fracture morphology.
Xu et al. [52]	Q690	Corrosion-fire coupled tensile behaviour	Coupled effects of corrosion, fire temperature and cooling method on post-fire tensile and fracture behaviour.

**Table 5:** Summary of studies on HSS beams and beam-columns.

Category	Authors	Steel Grade	Section Type	Loading Condition	Key Finding
Beams	Ma et al. [107]	700/900/1100 MPa	Cold-formed SHS, RHS and CHS	Four-point bending	High flexural resistance was obtained, while rotation capacity decreased with steel grade and slenderness.
Beams	Sun et al. [108]	S690	Welded I-section	Major- and minor-axis four-point bending	Local buckling governed flexure; prediction accuracy depended on section slenderness.
Beams	Zhang et al. [109]	S690	Press-braked channel section	Weak-axis bending	Bending direction and plate slenderness affected local buckling, moment resistance and codified predictions.
Beams	Wang et al. [110]	S960	Press-braked channel section	Four-point bending	Assessed codified resistance predictions and reported greater prediction variability for Class 3 and Class 4 sections.
Beams	Ding et al. [111]	340–1200 MPa advanced HSS	Lipped channel section	Four-point bending	Local-distortional interaction became critical; modified DSM rules improved prediction reliability.
Beams	Gong et al. [112]	Q460/Q690/Q960	HSS beams	Numerical lateral–torsional buckling study	Depth-to-width ratio and boundary condition governed lateral–torsional buckling resistance; steel grade had limited direct effect.
Beams	Li and Young [113]	700/900 MPa	Cold-formed SHS and RHS	Elevated temperatures up to 1000 °C	Material degradation reduced flexural capacity; existing fire-design provisions were assessed for SHS/RHS beams.
Beams	Ma et al. [114]	700–1100 MPa	Cold-formed SHS, RHS and CHS	Numerical flexural study	Validated numerical models were used for a broad slenderness range; codified methods and the DSM were assessed for HSS tubular beams.
Beams	Lan et al. [115]	HSS	CHS, EHS, SHS and RHS	Bending; tests and numerical analysis	The CSM was extended to HSS tubular sections and provided more accurate and less scattered resistance predictions than the examined DSM and codified methods.
Beam-columns	Yan et al. [116]	Q460C	Welded box section	Eccentric compression	Existing interaction formulae were conservative; modified formulae were proposed for HSS box sections.
Beam-columns	Ma et al. [117]	Q690	Welded H-section	Eccentric compression; minor-axis bending	Specimens failed mainly by minor-axis flexural buckling with yielding, and the examined European design method provided accurate predictions.
Beam-columns	Ma et al. [118]	Q690	Welded H-section	Numerical parametric study	Parametric analyses extended the Q690 test data and supported design assessment for compression-bending interaction.
Beam-columns	Ma et al. [119]	700/900 MPa	Cold-formed SHS and RHS	Combined compression and bending	Section slenderness and loading eccentricity controlled resistance; 51 tests were used for design evaluation.
Beam-columns	Su et al. [120]	S960	Welded I-section	Compression and minor-axis bending	Codified interaction curves gave conservative and scattered predictions because of conservative bending end points.
Beam-columns	Ma et al. [121]	S700	Built-up I-section from two channels	Short and long eccentric compression tests	The prediction trends differed between short and long members, and modified interaction equations were proposed.
Beam-columns	Yang et al. [122]	Q1100	Welded I-section	Beam-column tests and numerical parametric study	Current methods were not fully calibrated for Q1100 UHSS; an improved approach was proposed.
Beam-columns	Wang et al. [123]	Q460C	Welded H- and box sections	Constant axial load and cyclic lateral loading	Compact sections showed good energy dissipation; component plate slenderness controlled cyclic deterioration.
Beam-columns	Hai et al. [124]	Q690D	Compact welded H-section	Strong-axis cyclic loading	Plastic local buckling dominated failure; compact sections showed favourable ductility and energy dissipation.
Beam-columns	Hai et al. [125]	Q690	H-section beam-column	Cyclic numerical simulation	Ductile fracture and cyclic softening improved simulation of hysteresis and failure modes.
Beam-columns	Yang et al. [126]	Q690	Welded box section	Constant axial force and cyclic lateral loading	Base local buckling governed deterioration; the examined European cross-section classification was evaluated against the cyclic response.
Beam-columns	Chen et al. [127]	Q690	Thin-walled welded H-section	Weak-axis cyclic loading	Base local buckling governed failure; axial load ratio and flange slenderness controlled response.
Beam-columns	Sun et al. [128]	Q960	Welded I-section	Post-fire combined compression and major-axis bending	Residual resistance depended on temperature, eccentricity and slenderness; improved interaction curves were proposed.

**Table 6:** Key parameters and reported design findings for selected HSS connections.

References	Connection Type	Steel Grade and Investigated Parameters	Key Finding
Wang et al. [166]	Slip-critical bolted connection	Q550, Q690 and Q890; shot-blasted surfaces	Mean slip factors were 0.502, 0.457 and 0.455; a uniform design value μ = 0.40 was recommended
Lan et al. [168]	CHS X-joints	S700, S900 and S1100; 0.2 ≤ β ≤ 1.0, 10 ≤ 2γ ≤ 50, 0.5 ≤ τ ≤ 1.0; brace axial compression	Mean predicted/FE ratios for EN/CIDECT increased from 1.08/1.00 for S700 to 1.32/1.23 for S1100; predictions became increasingly unconservative above S700
Lan et al. [169]	RHS X-joints	S460, S690 and S960; β, 2γ, chord preload	CIDECT prediction became increasingly unconservative with increasing steel grade and 2γ, and decreasing β for chord-face plastification
Lan et al. [17]	CHS T/X-joints	Q960; geometry ratio and HAZ effects	Existing equations required evaluation considering steel grade, HAZ and failure mode
Pandey and Young [170,171]	RHS/CHS T-joints	S900/S960 cold-formed tubular joints; β, τ, 2γ	Current provisions showed limited accuracy for UHSS tubular joints
Pandey and Young [172]	Tubular X-joints	S900/S960; 0.34 ≤ β ≤ 1.0, 0.53 ≤ τ ≤ 1.28, 20.2 ≤ 2γ ≤ 38.9	Existing provisions did not provide sufficiently accurate predictions for UHSS X-joints

## Data Availability

No new data were created or analyzed in this study. Data sharing is not applicable to this article.
